# SH-IDS: a resilient self-healing intrusion detection framework against DoS and DDoS attacks in IoT systems

**DOI:** 10.1038/s41598-026-48932-2

**Published:** 2026-04-28

**Authors:** Mahawish Fatima, Osama Rehman, N. Z. Jhanjhi, Saqib Ali

**Affiliations:** 1 Department of Software Engineering, Bahria University Karachi Campus, Karachi, Pakistan; 2 School of Computer Science, Taylor’s University, Subang Jaya, Malaysia; 3 Office of Research and Development, Asia University, Taichung, Taiwan; 4 Department of Information Systems, Sultan Qaboos University, Muscat, Oman

**Keywords:** Internet of things, Intrusion detection, IoT security, Self-healing, Machine learning, Energy science and technology, Engineering, Mathematics and computing

## Abstract

The rapid proliferation of Internet of Things (IoT) devices and advanced obfuscation techniques has led to increasingly sophisticated Denial of Service (DoS) and Distributed Denial of Service (DDoS) attacks. Traditional intrusion detection systems (IDS) without adaptive capabilities often fail to detect such attacks, resulting in a rising success rate of DoS/DDoS incidents each year. To address these limitations, this study proposes a self-healing intrusion detection system (SH-IDS), a machine learning (ML)-based IDS that enhances IoT network security through collaborative and autonomous adaptation. Unlike existing solutions, the threshold values in SH-IDS are adaptive and computed based on IoT device configurations. When an IoT device detects resource usage exceeding these thresholds, it sends a danger signal that triggers a self-healing process to update the detection model, thereby blocking malicious traffic. Six ML algorithms are evaluated to assess SH-IDS performance. Results show that Random Forest (RF) achieves 59.6% accuracy before self-healing and 99.9% after self-healing, with the highest true positive and true negative rates in detecting emerging DoS/DDoS attacks. Overall, the findings demonstrate that SH-IDS is an effective, adaptive, and flexible solution for mitigating both existing and emerging DoS/DDoS threats.

## Introduction

The Internet of Things (IoT) environment has expanded significantly due to the increasing number of devices, sensors, and actuators. Autonomous devices collect, process, and transmit information over the Internet; therefore, IoT has found its place as essential part of today’s technological network^1^. The global growth of IoT devices continues to expand, and according to estimates, the number of IoT devices that will be connected globally by 2030 is approximately 29.4 billion, with a value of nearly $621.6 billion^2^. Despite their advantages, including cost-effective mass deployment and broad technological impact, the devices and systems are extremely susceptible to cyberattacks. Typical IoT devices have limited computing power, low security controls, weak firmware update strategies, and weak encryption. These limitations make IoT systems profitable targets for malicious perpetrators seeking unauthorized access or interference through various attacks including information leakage, Denial of Service (DoS), and Distributed Denial of Service (DDoS)^3^.

Recent events show the increasing volume and complexity of DoS/DDoS attacks either by compromising IoT devices or on IoT networks. The 2017 BrickerBot attack proved the crippling potential of such attacks by permanently disabling IoT devices^4^. Similarly, China’s massive 2018 DDoS attacks hijacked domestic appliances, routers, and IP cameras^5^. Moreover, in 2025, Cloudflare reported a record-breaking DDoS attack named Aisuru that targeted telecommunication providers with 29.7 Terabits per second (Tbps)^6^.

Due to the increasing complexity and diversity, mitigating such attacks has become a challenge for the research community. Consequently, Machine Learning (ML) based Intrusion Detection Systems (IDS) have gained considerable attention in identifying the anomalies and detecting the flood of traffic generated by DoS/DDoS attacks^3,7^. These ML-based IDS achieve very high accuracy in laboratory settings. However, their effectiveness decreases in real-world networks^8^. Since most of the researchers have not tested the IDS in real world scenarios or on unseen data, their models are likely to produce high false positive rate or even do not capture attacks, particularly those attacks employing evasion methods such as IP spoofing, encryption, packet fragmentation, and protocol tunneling. These evasion methods allow attackers to escape detection of traditional ML-based IDS and persistently invade IoT environments.

The robustness of IDS models has been discussed in recent studies by introducing a self-healing mechanism that has the ability to support autonomous detection, reaction, and healing against security assaults^9^. Some of the researches including^10–12^, have presented self-healing IDS; however, their proposed mechanisms are still limited in terms of flexibility. Moreover, the existing self-healing IDS solutions predominantly use fixed threshold values in an attempt to distinguish between benign and malicious traffic, which may fail to detect in dynamic IoT environments. In addition, the healing process is also prone to performance overhead and operational disservice. Consequently, raising system downtime that subsequently leads to overall performance degradation of the IDS model.

The exponential growth in DoS/DDoS attacks and limitations of existing solutions necessitate an adaptive, autonomous, and resource-efficient approach that can effectively detect novel attacks within IoT environment. This study proposes a ML enabled self-healing intrusion detection system (SH-IDS) for dynamic IoT environment that addresses these challenges. The proposed framework also includes a health monitoring module to trigger the self-healing process, allowing timely detection and mitigation of DoS/DDoS attacks. In addition, the system dynamically updates the IDS model based on newly observed data, ensuring efficient resilience against evolving attack patterns with minimal system resources.

### Contribution

In this paper, we present a novel DoS/DDoS detection framework for an IoT network while highlighting the role of individual IoT nodes to initiate a self-healing process to achieve efficient DoS/DDoS detection. Therein, we present the design and development of DoS/DDoS detection framework that utilizes six ML classifiers, including Random Forest (RF), Decision Tree (DT), K Nearest Neighbor (KNN), Gradient Boosting (GB), Naive Bayes (NB), and Neural Network (NN) as detection components based on their widespread adoption in the research community. We also present an implementation and evaluation of the proposed framework utilizing two of the publicly available datasets, namely ToN_IoT^13^ and BoT-IoT^14^, with in-domain as well as cross-domain scenarios. Specifically, the research presents the following key contributions:

- This study provides a critical insight into the recent state-of-the-art studies in self-healing IDS. A comparative analysis highlights the strengths, limitations, and gaps in existing approaches. These findings emphasize further investigation to design an adaptive and resilient IDS to protect IoT-based systems.
- This study presents a novel framework, *SH-IDS* (Self-Healing Intrusion Detection System), an ML-enabled IDS with self-healing capability to detect existing and emerging DoS/DDoS attacks. The self-healing mechanism employs a dynamic, trigger-based adaptive process that updates the IDS model when IoT devices experience resource depletion.
- The proposed *SH-IDS* is evaluated across six ML classifiers using two scenarios: in-domain testing with the TON_IoT dataset and cross-domain testing with the BoT-IoT dataset to assess generalization and adaptability in varied network environments, which are lacking in state-of-the-art studies. Furthermore, the framework’s performance is evaluated before and after the self-healing process to assess its adaptive learning capability.
- To evaluate the performance of all ML models multiple metrics such as accuracy, parameters derived from the confusion matrix including True Positive Rate (TPR), True Negative Rate (TNR), False Positive Rate (FPR), and False Negative Rate (FNR), training/testing time, memory usage, and CPU usage are used during train and test phases.

The rest of the paper is organized as follows: Section 2 provides an overview of IDS and Datasets. Section 3 provides a critical review of existing self-healing IDS research. Section 4 introduces the proposed SH-IDS framework and provides an in-depth discussion along with details of the rigorous experimentation process and threshold calculation. Section 5 presents the experimental scenarios, results, and discussion of the in-domain, cross-domain, and after self-healing analyses. Finally, Sect. 6 concludes the paper and highlights potential future research directions.

## Background

### ML-enabled IDS

An IDS is a tool designed to continuously monitor networks or host systems for malicious activities or policy violations. Upon detection, an IDS generates an alert that can be logged or sent to a security analyst for further investigation^15^. IDS are categorized based on their scope: Network-based IDS (NIDS) watches over network entry points, Host-based IDS (HIDS) protects important systems from security breaches, and Hybrid IDS combines both types. There are also two types of IDS: anomaly-based and signature-based. Anomaly-based IDS sets a standard for normal behavior and uses ML to encounter deviations. Signature-based IDS, on the other hand, uses predefined attack signatures to detect attacks^16^. Anomaly-based IDS uses ML algorithms to detect malicious activities in networks. An ML-enabled IDS requires datasets with both legitimate and illegitimate samples. IDS that use ML and both supervised and unsupervised learning algorithms have been developed to detect new attacks in IoT networks^17^. The model is trained on labeled datasets for supervised learning. However, in unsupervised learning models, labeled data are not required. But a major problem with current IDS solutions is that they generate many false alarms, which can disturb the normal network operations; moreover, existing IDS are inefficient to fight against state-of-the-art obfuscation techniques^18^.

### Self-healing

Self-healing is a term used in many fields, such as biology, materials science, and computing. It is the ability of a system to show resilience and recover from unexpected events^9^. Self-healing encompasses an automatic recovery process in which a system identifies, diagnoses, and corrects faults, without human intervention. The self-healing system primarily lies in its ability to enhance system dependability by minimizing downtime and maintaining operational stability despite the occurrence of faults. Self-healing in IDS refers to an automated, adaptive mechanism in which the IDS upgrades its detection capabilities for previously unseen or novel attacks. In the context of IoT-based DoS/DDoS detection, the self-healing mechanism is activated when the system identifies abnormal/suspecious device-level behavior that includes resource exhaustion, increased packet rates or sizes, or degraded performance. This indicates that the model has not been able to identify the DoS/DDos attack. By initiating the self-healing process, the IDS model starts a repair cycle by retraining and updating the existing detection model. Consequently, making the model adaptable to emerging threats without manual intervention.

### Datasets

This section describes the dataset applied to train the ML-IDS model in this study. The discussion is based on the TON_IoT and BoT_IoT datasets to demonstrate their usefulness for in-domain and cross-domain testing, respectively. Both datasets have been widely utilized in the development and assessment of ML-based IDS. These datasets being incorporated in the proposed IDS makes it possible to generalize our IDS for the heterogeneous IoT environments

#### TON_IoT dataset

The TON_IoT dataset^19^ contains telemetry data gathered from various IoT network devices. TON_IoT contains records for both benign and malicious behavior sessions from IoT testbed. The testbed includes ten IoT devices, including weather sensor, Temperature and Humidity sensors, Modbus sensor, Motion Light, Thermostat, Smart fridge, Garage Door, GPS Tracker, Smartphones, Smart TV. The TON_IoT dataset is available in both CSV and PCAP formats, covering multiple attack types such as DoS/DDoS, XSS, password cracking, reconnaissance, MITM, ransomware, backdoors, and SQL injection. The dataset has four directories: Windows 7, Windows 10, Network, and Win10–Network with varying sizes: 28,366 (132 features), 35,975 (124 features), 21,978,632 (45 features), and 1,073,754 records, respectively. This research focuses on the binary classification of DoS/DDoS and normal traffic. Due to class imbalance, a class-weighting strategy is applied to ensure balanced learning, and improving detection performance across uneven sample distributions.

#### BoT-IoT dataset

The Bot-IoT^14^ is a labeled and publicly available dataset generated from IoT testbed. The BoT-IoT dataset contains 72 million records, for benign and attack category; each record has 46 unique features providing information for each record. BoT-IoT is on-shelf dataset and available in both PCAP and CSV formats. Normal traffic is generated using Ostinato, whereas attack types, including DoS, DDoS (HTTP, TCP, UDP), port scans, keylogging, and data exfiltration are simulated on four Kali Linux machines. The dataset testbed is based on IoT devices communicating via MQTT through Node-RED middleware, representing various scenarios, such as a weather station, smart fridge, motion-activated lights, garage door, and thermostat.

## Recent studies in self-healing

This section presents critical insights into the state-of-the-art self-healing mechanism in an IDS. The self-healing capability in IDS solves the challenging detection of emerging and state-of-the art attacks, enabling systems to detect, diagnose, and recover from disruptions automatically. A self-healing IDS can operate in real-world scenarios, maintain normal operations and reduce the impact of an assault.^20^.

This study^10^ introduced an innovative self-healing methodology to improve cloud computing environments through the application of Recurrent Neural Networks (RNNs) combined with Long Short-Term Memory (LSTM). The IDS model uses both past and present data to accurately predict failures and a performance 95%, with a false positive rate of 3%. This system has proactive fault detection and mitigation. But retraining RNN and LSTM is often difficult and requires considerable computing power, which makes it less practical for IoT-based systems that do not have many resources.

A recent study^21^ presented the SHARP-Net. The proposed framework has three main parts, including an IDS, an Intrusion Mitigation System (IMS), and an Alert Management System (AMS). The proposed IDS analyze the network logs upon detection and, sends alerts to IMS through AMS. This coordination allows the IMS to change the network and cut off PMUs that have been compromised. The IDS of SHARP-Net depends on predefined rules, which makes it less flexible for emerging attacks.

**Table 1:** Comparison of existing Intrusion Detection Systems (IDS) with self-healing capabilities across different domains.

Paper	Domain	Autonomous	ML	Accuracy (%)	IDS type	Adaptive	Resources required in self-healing		
							Time (s)	Memory (MB)	CPU (%)
This paper	IoT	Fully	Yes	99.9	NIDS	Yes	28.12	2280	49
^21^	IoT	Fully	No	95.3	NIDS	No	–	–	–
^10^	Cloud Computing	Fully	Yes	95	HIDS	No	–	–	–
^22^	IoT	Fully	Yes	97	HIDS	Yes	–	–	–
^11^	WSN	Fully	Yes	90	NIDS	No	18	–	–
^23^	IoT	Fully	No	99	NIDS	No	–	–	–
^9^	IoT	Fully	Yes	94	HIDS	No	–	–	–
^24^	IoT	Partially	No	83	NA	No	–	–	–
^25^	Cloud Computing	Fully	No	91.5	HIDS	No	–	–	–
^26^	Network Security	Partially	No	88	NIDS	No	–	–	–
^27^	VANETs	Fully	No	85	NIDS	No	–	–	–

The authors of^22^ proposed a hybrid IDS that integrates signature-based and anomaly-based detection to enable self-healing capabilities. Signature detection identifies known attacks. Furthermore, the detected anomalies are analyzed using LSTM-RNN, are converted into new signatures. The Evaluations of the proposed hybrid IDS carried out using the UNSW-NB15 and ADFA-LD datasets showed that the C5 classifier achieved a 97% detection rate with an 8% false positive rate, whereas LSTM-RNN achieved a 90% detection rate with a 17% false alarm rate. Despite its high detection rate, this approach remains vulnerable to obfuscated attacks and suffers from a relatively high false alarm rate during anomaly detection, which can lead to inefficient resource utilization.

Another study^9^ proposed a framework for an IoT-based system to recover from faults and cyberattacks. The framework has three parts: a Host-based IDS to find problems, a Health-Monitoring Module to check how well devices are working, and an Autoremediation Module to take corrective action based on pre-trained neural networks and evolutionary algorithms. The framework was tested on IoT devices in situations such as DoS and process exploitation. This showed that it could adapt well, and the more devices that joined the network, the more accurate the remediation became. The study is limited to HIDS, and does not cover attacks that affect the entire network or come from more than one source such as DDoS. Additionally, the results are mainly discussed qualitatively, without any quantitative metrics, which makes it difficult to judge the performance.

The study^23^ presented a self-healing mechanism for an IoT system. The framework was built using an AMI that contains a layered architecture. In proposed framework, an IDS consists of three components to produce predictions, including Sensing Approach for keeping an eye on IoT parts, Awareness of Issues for finding problems, and Responsibility and Actions for taking care of problems, and corrective actions. The experimental results show that the proposed self-healing mechanism achieves 99% accuracy. But using fixed thresholds, such as a 50% battery level, without testing them in real life makes it harder to adapt to different IoT environments.

In paper^25^, an agent-based network model was presented for vulnerability detection, classification, and remediation. The framework uses the third-party tool Nessus to scan for vulnerabilities, whereas centralized database to manage patches to overcome the discovered vulnerabilities in system. This allows the system to automatically detect and apply missing updates. The proposed framework is efficient as it reduces security risks. However, the proposed framework is specifically designed to detect Windows 7 system vulnerabilities, which makes it less suitable for other platforms.

R. Seiger et al.,^24^ proposed a framework to enhance resilience in distributed IoT processes using a MAPE-K feedback loop. Built on the PROtEUS engine that incorporates context-aware self-healing to monitor IoT devices, detect errors, and plan actions. The proposed framework improves fault tolerance by deploying tasks to alternative devices. However, the high computational demands on super-peer nodes limit its feasibility in resource-constrained IoT environments.

The paper^26^ presented an IDS with a self-healing mechanism. The proposed framework is based on the human immune system for detecting botnets. An IDS has a central server for managing the system, client agents for finding and fixing infections, and a quarantined network to protect systems. The IDS classifies and isolates compromised hosts, and requires minimal assistance from users. Proposed IDS was evaluated for IRC (RxBot) and HTTP (WarBot) botnets. However, the IDS decision mainly depends on the static configuration, which may limit how well it works for systems based on heterogeneous IoT devices.

In^27^, a framework based on biology that combines an artificial immune system (AIS) to find intrusions with self-healing capabilities was presented. This model is based on idiotypic networks in biological immune system. The proposed IDS differentiates between “self” (normal) and “non-self” (anomalous) behavior. When an intrusion is found, the self-healing mechanism automatically determines and fixes the problem by changing the network structure or fixing the configurations. However, the study di not yield quantitative evaluation results, thereby constraining the empirical validation of the proposed framework.

Table 1 presents existing IDSs with self-healing capabilities. The table highlights that several studies employ ML to enhance anomaly detection, such as^9,10^, and^11^; however, most of these approaches lack the self-adaptive mechanisms required for dynamic response and performance optimization. In addition, very few studies provide detailed evaluations of resource requirements, such as time, memory, and CPU utilization, during self-healing operations, which are critical factors for IoT deployment. With the exception of a few works (e.g., in existing study^10^), most approaches emphasize theoretical or qualitative assessments, offering limited insight into real-time feasibility. Moreover, most existing studies rely on fixed threshold values without discussing the rationale behind their selection.The proposed method addresses these gaps by offering a lightweight, interpretable, and resource-efficient self-healing IDS designed for real-world IoT environments.

## Proposed ML-enabled IDS with self-healing capability

**Fig. 1:**
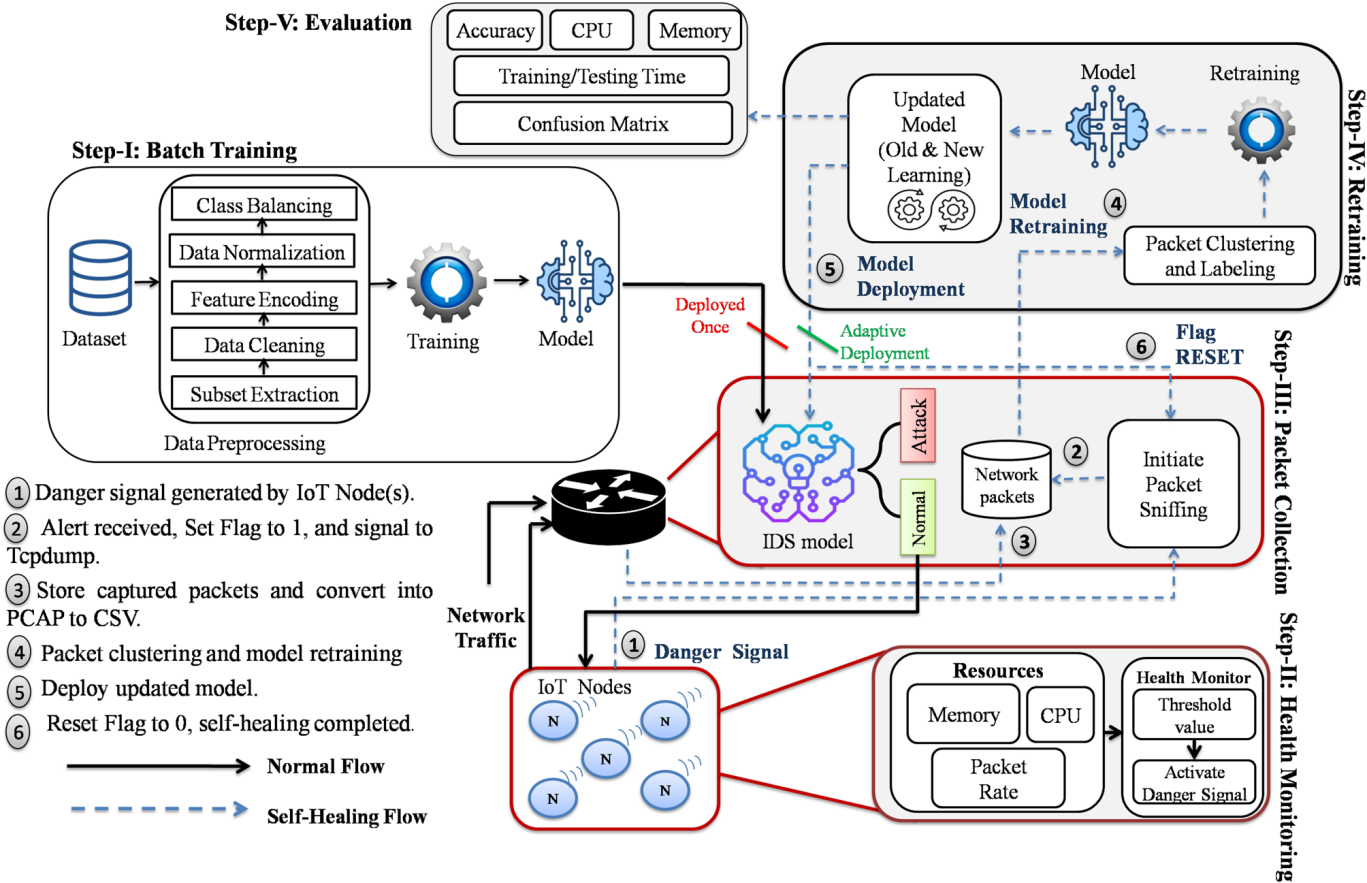
An overview of the proposed SH-IDS framework.

This section presents the insights into the proposed SH-IDS framework. The proposed SH-IDS framework Illustrated in Fig. 1, consists of five sequential step including Batch Training, Health Monitoring, Packet Collection, Retraining, and Evaluation. In the first stage, the dataset is preprocessed and applied to ML classifiers to train the intrusion detection model. In the second stage, the health module monitors the resource consumption of IoT devices and generates danger signals upon excessive usage. The third stage receives danger signals and perform packet collection. In the fourth stage, network packets are clustered, and use in training ML model. Finally, the fifth stage evaluates the overall effectiveness and adaptability of the proposed self-healing mechanism.

### Step I: batch training

In the proposed SH-IDS, Step I includes several phases used to train the initial ML model. First, batch training requires the dataset; the dataset is supplied to the data preprocessing phase, which further consists of five processes, including, subset extraction, data clean, feature encode, normalization, and class balance. This study primarily targets DoS/DDoS attacks; therefore, the subset extraction phase selects records related to DoS/DDoS traffic along with normal samples, which are then processed during the data cleaning sub-step. The data cleaning process removes the redundant and empty records, handles missing values, and mitigates outliers to ensure data quality and integrity. The feature encoding process transforms the categorical data into a numerical form, enabling more effective ML model training. In this study, the label encoding technique has been employed for feature encoding to transform the categorical features into numerical values where it assigns a unique integer value to each category in a column. In this study, data normalization is performed via the min–max method to scale numerical features within the range [0, 1]. Since SH-IDS is designed for binary classification, class balancing is applied to have an equal number of samples in the attack and normal classes. We have employed the undersampling to obtain equal numbers of samples from both classes. After data preprocessing, the processed data is split into training and testing samples. During the training phase, the training samples are fed into the ML classifiers. The performance of the trained model is then evaluated using the testing samples. After evaluation, the trained model is deployed in a real-world network. Once deployed, the model continuously monitors the network traffic to detect anomalies. It is noteworthy the model is deployed only once during the initial setup.

### Step II: health monitoring

IoT devices possess limited resources, such as CPU and memory, which are sufficient for normal operation^28,29^. When an attacker launches a DoS or DDoS attack, they overwhelm these resources using a surplus of packets with fake requests. Computational resources are exhausted, and the device becomes inaccessible or unresponsive to the legitimate user. Monitoring of resource consumption allows detection of abnormalities and is an early warning of potential attacks^30^.

Given these limitations, the framework presented in Fig. 1 accommodates two significant modules in Step I, which are embedded in the IoT device. The first step calculates the CPU, memory, and packet rate thresholds by monitoring the device behavior during normal operation and under attack conditions during the system setup. As this study focuses on network-layer DoS/DDoS attacks, packet rate is taken as a significant metric along with CPU and memory. The second module compares the actual resource usage with predefined threshold values. As soon as the CPU, memory, or packet rate exceeds these thresholds, the health monitor generates a danger signal. The resource-centric danger signal triggers the self-healing process described in Fig.  1 and follows steps 1 through 6.

#### Calculation of threshold value

The following sections describe the approach used to calculate the threshold values. These values are computed through extensive experiments. Virtual Machines (VMs) are employed to simulate IoT devices based on the specifications given in Table 2. Additionally, to perform a realistic analysis of the workload, a Python script is used to simulate the VM as a smart temperature sensor that periodically transmits temperature readings to the IoT gateway. We observed that under typical conditions, the IoT node uses approximately 4–5% CPU and 36–38 MB of memory to process four incoming and outgoing packets per second (pps).

**Table 2:** Configuration details of the smart temperature sensor device.

Resource	Configuration
Device name	Smart Temperature
Memory	512 MB
Processor	Single-core, ARM architecture, 1.2 GHz
Operating system	Raspbian Lite

**Fig. 2:**
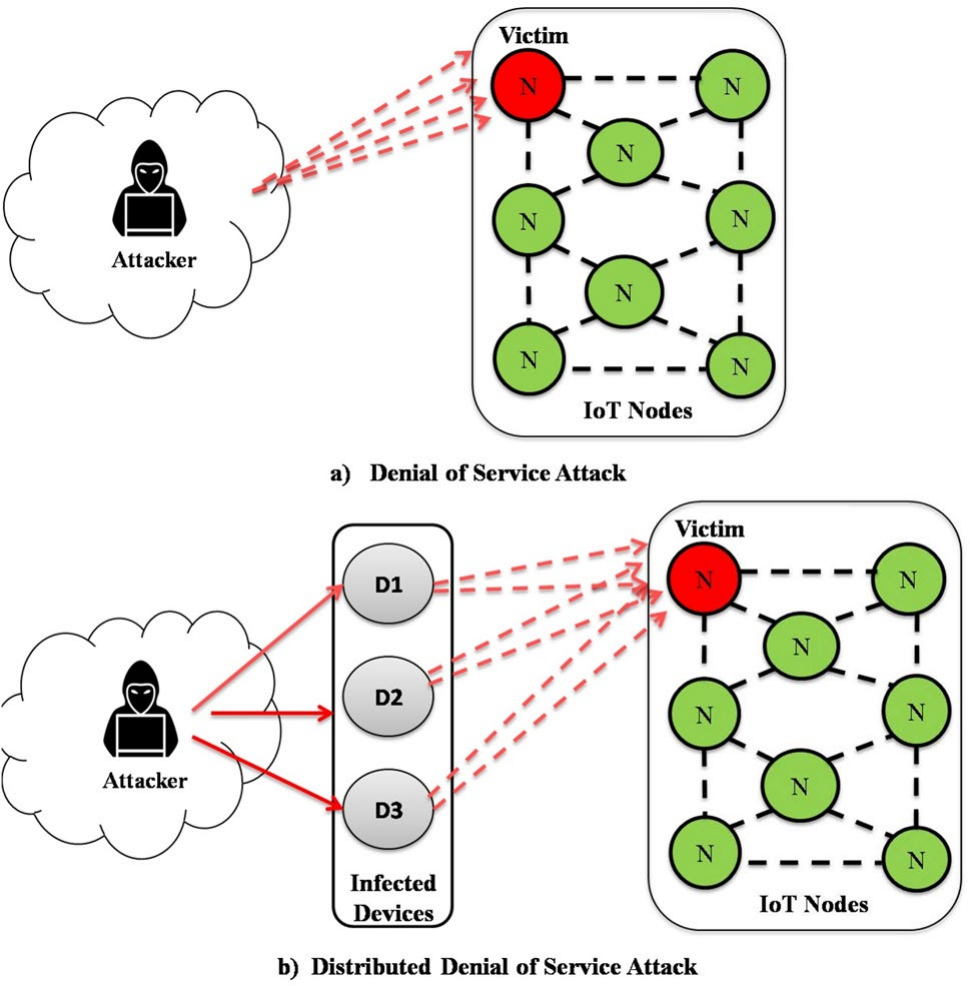
DoS and DDoS Topology.

**Fig. 3:**
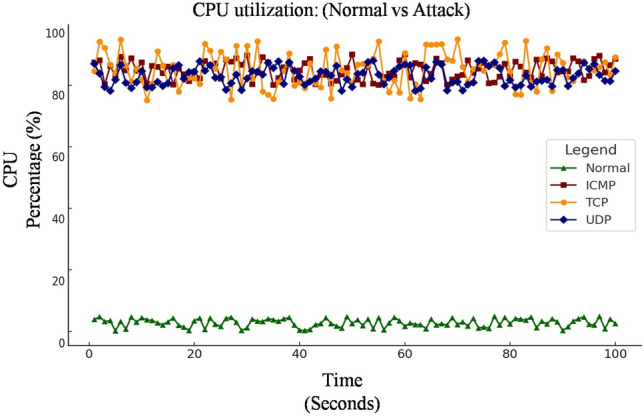
CPU usage in normal and attack scenarios.

**Fig. 4:**
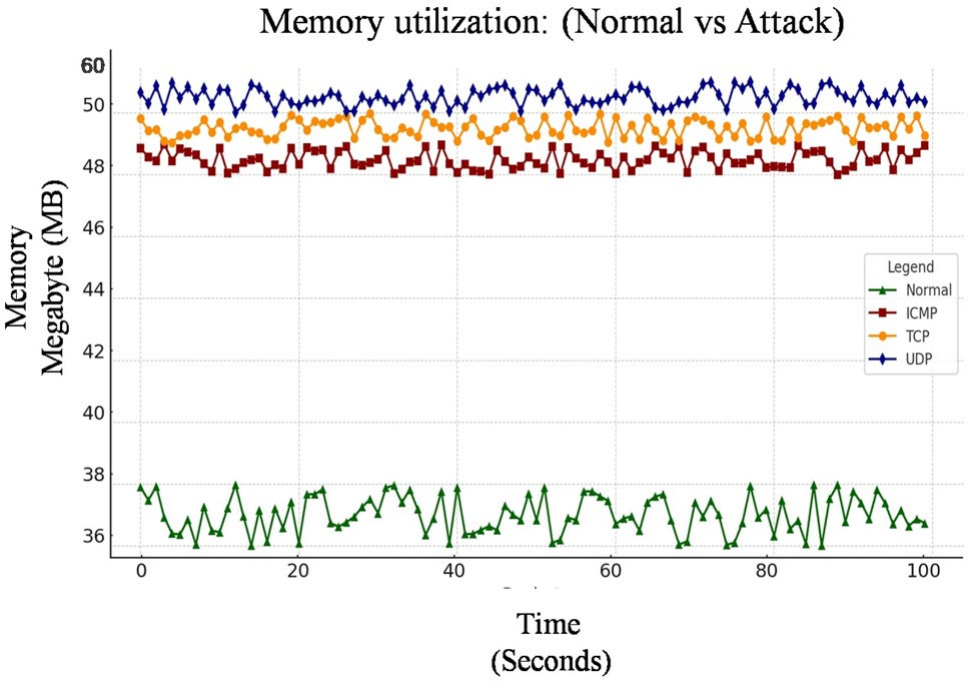
Memory utilization in normal and attack scenarios.

**Fig. 5:**
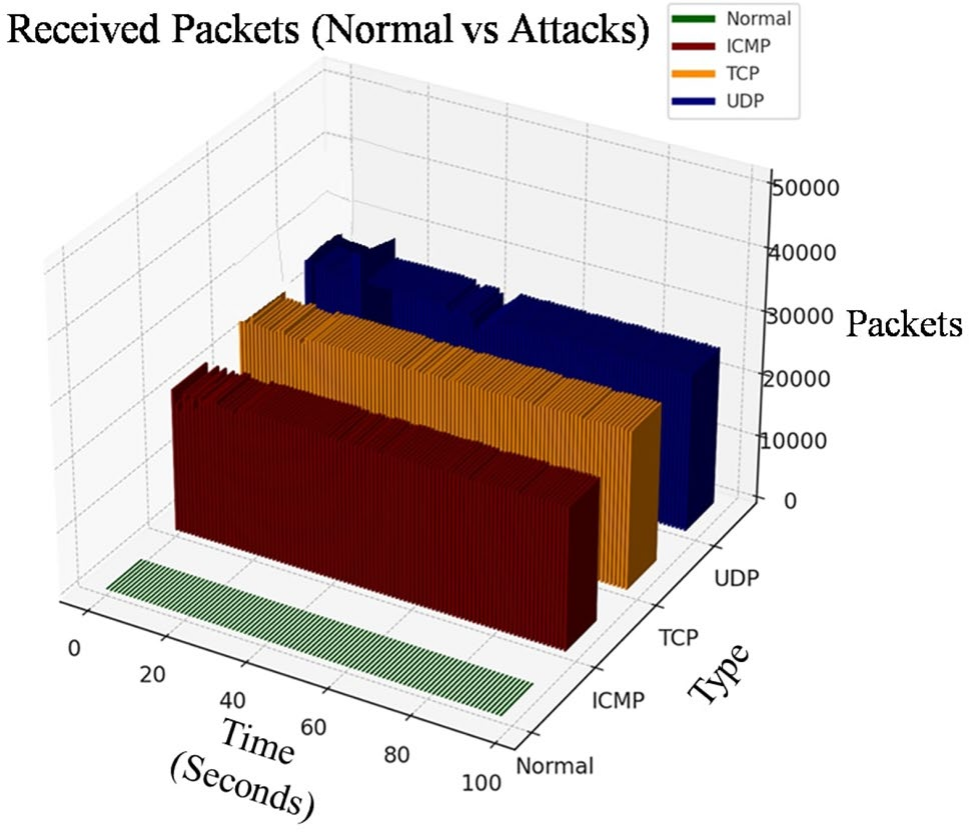
Packets received in normal and attack scenarios.

To observe resource consumption in IoT devices. We have used the topologies shown in Fig. 2 to launch DoS and DDoS attacks. In this regard, tools including HPING3^16^, Metasploit^31^, and LOIC^32^ are used on Kali Linux, which generate ICMP, UDP, and TCP floods to target IoT devices. During the attack, we observed significant resource consumption in the IoT device as shown in Figs. 3, 4, 5. These figures comprehensively represent the IoT devices in two states: one normal state and an attack state.

As shown in Fig. 3, the CPU utilization of the IoT device ranges from 1.7% to 4.4% under normal conditions, including data collection, communication, and transmission. However, during attack events, the CPU utilization increases significantly, ranging from 79% to 89%. The CPU threshold value is 41.7% calculated using Equation (1), where CPU_max represents the maximum CPU utilization during normal operation and CPU_min represents the minimum CPU utilization observed during an attack event. The corresponding prediction is provided in Equation (2).

As shown in Fig. 4, the IoT device consumes 37.9 MB of memory under normal conditions and uses at least 48.3 MB of memory during an attack. The threshold value for the memory based on the IoT device is calculated as 43 MB using Equation (3). Furthermore, the corresponding prediction is expressed in Equation (4).

1 $$\begin{aligned} T_{CPU}&= \frac{CPU_{\text {max}} + CPU_{\text {min}}}{2} \end{aligned}$$

2 $$\begin{aligned} \text {Prediction}&= {\left\{ \begin{array}{ll} \text {Normal}, & \text {CPU}_{\mathrm{usage}} < T_{CPU} \\ \text {Attack}, & \text {CPU}_{\mathrm{usage}} \ge T_{CPU} \end{array}\right. } \end{aligned}$$

3 $$\begin{aligned} T_{memory}&= \frac{Memory_{\text {max}} + Memory_{\text {min}}}{2} \end{aligned}$$

4 $$\begin{aligned} \text {Prediction}&= {\left\{ \begin{array}{ll} \text {Normal}, & \text {Memory}_{\mathrm{usage}} < T_{\text {memory}} \\ \text {Attack}, & \text {Memory}_{\mathrm{usage}} \ge T_{\text {memory}} \end{array}\right. } \end{aligned}$$

In addition to the threshold calculation, as shown in Fig.  5, $\text {packet}_{\text {max}} = 5$ is the packet rate observed during normal operating conditions, and $\text {packet}_{\text {min}} = 19{,}681$ is the rate observed during the event packet. This yields a threshold value of $T_{\mathrm{packet\_rate}} = 9{,}843$ pps.

5 $$\begin{aligned} T_{packet\_rate}&= \frac{Packet_{\text {max}} + Packet_{\text {min}}}{2} \end{aligned}$$

6 $$\begin{aligned} \text {Prediction}= {\left\{ \begin{array}{ll} \text {Normal}, \ \text {Packet \ Rate} <  T_{packet\_rate} \\ \text {Attack}, \  \text {Packet \ Rate} \ge T_{packet\_rate} \end{array}\right. } \end{aligned}$$

The derived threshold values are provided as input to the health module, which continuously tracks system resources and issues an alert whenever a resource exceeds its respective threshold. The resource monitoring steps are detailed in Algorithm 1. The module uses the psutil library to obtain the latest state of each resource and compares the values against the provided threshold, represented by $T$. When the observed CPU, memory, and packet rate exceed their respective thresholds, an alert is triggered, thus activating the self-healing process. When the system initiates the self-healing process, the IoT device, on the other hand, protects itself by terminating processes that consume excessive CPU resources (more than 41.7%), thereby preventing a potential failure of the IoT device.

**Algorithm 1:**
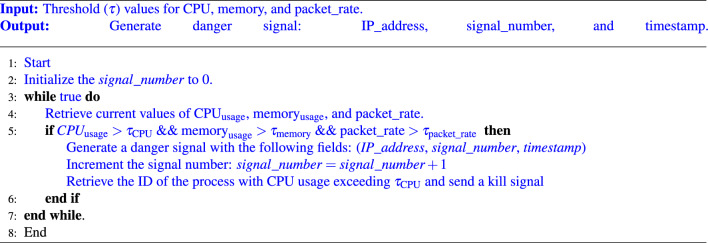
Resource Monitoring and Danger Signal

### Step III: packet collection

After the danger signal is generated and received by the packet collection module, the packet sniffing module starts the process given in Algorithm 2. The algorithm obtains important information from the danger signal, such as the IP address of the IoT device that generated the danger signal, the signal_number, and the time. Then, the module instructs the packet sniffer (tcpdump.exe) to capture the network traffic and sets the variable Flag to “1”, which indicates that the self-healing process is enabled.

**Algorithm 2:**
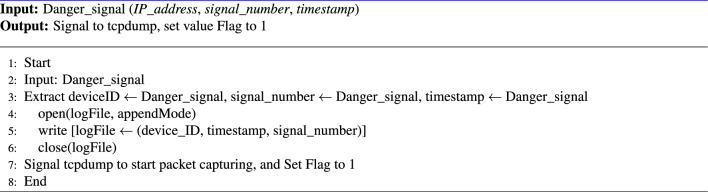
Receiving Danger Signal and Initiate Packet Capturing

According to studies, such as^33,34^ data samples in the range of 70,000 to 80,000 are sufficient to effectively train an IDS that accurately discriminates between malicious and normal traffic. Considering these studies, we terminated the packet capture after recording 74,208. The captured packets are saved in a PCAP file by default. The PCAP file is then converted to CSV format after the steps of Algorithm 3, to facilitate clustering and model retraining.

**Algorithm 3:**
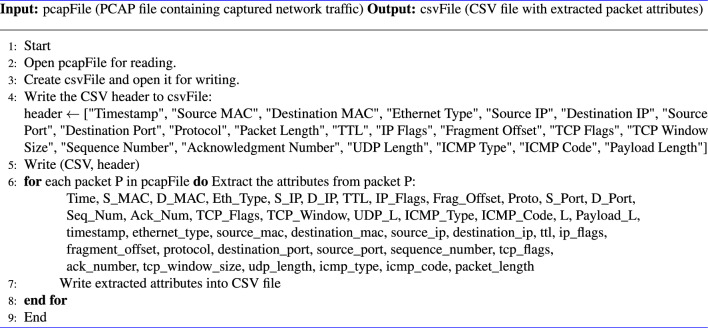
Convert PCAP File to CSV with All Packet Attributes

### Step IV: retraining

In this study, retraining is performed at the IoT gateway (Edge layer), which has sufficient resources to execute the retraining process with lower latency compared to the Fog and Cloud layers^35^, thereby reducing downtime and maintaining the overall stability of the system. To obtain labeled data, the captured network packets are fed as input to the K-means clustering algorithm. It is an unsupervised ML method, widely used in the research community for clustering and labeling unlabeled data^36^. K-means efficiently divides unlabeled data into distinct groups by identifying similar features and labeling them based on a parametric configuration. The parameters and configurations used in this study are summarized in Table 3.

Here, the K-means++ method is used to determine the initial centroids^37^ within the collected network packets. The K-means++ method strategically selects centroids to improve the convergence speed and the stability of the clustering. The algorithm performs approximately 300 iterations per run to achieve better convergence. Furthermore, the timestamp feature of the threat signal is used to assign a label using the k-means++ method. Packets captured outside the threat signal period are classified as normal, while packets captured during these periods are identified as attack traffic. This temporal validation ensures that the “normal” cluster accurately represents benign network traffic and the attack clusters accurately represent all malicious patterns.

**Table 3:** Configuration parameters for K-means clustering.

Parameter	Value
n_clusters	2
init	K-means++
max_iter	300
n_init	10
algorithm	elkan

Furthermore, the labeled dataset is used to retrain the ML model by incorporating new knowledge with prior learning ensuring retention of prior inference knowledge while incorporating emerging attack patterns, and the updated model is integrated with the existing IDS, which enhances the IDS’s ability to detect current and emerging DoS/DDoS attacks. Furthermore, a signal is sent to the packet sniffing module to reset the Flag variable to 0, indicating that the self-healing process has been completed. The updated system then resumes normal monitoring through the alert receiver module and is ready to respond to future threat signals. The performance of the retrained IDS model is subsequently evaluated in the following stage.

### Setp V: evaluation

In this section, the evaluation parameters are discussed. Accuracy and the confusion matrix are well-known and widely used evaluation metrics. However, for training and testing time, as well as memory and CPU utilization, we have proposed our own evaluation measures, which are described below.

**Training Time:** It is the time required to perform multiple sub-tasks before model training. In the self-healing process, the training time consists of data preprocessing, clustering (K-means), and the time required in retraining ML models, calculated by using Equation (7). Given that $\text {Time}_{\text {Clustering}}$: Time required for labeling samples using K-means clustering, $\text {Time}_{\text {Preprocessing}}$: Time required for data preprocessing tasks and $\text {Time}_{\text {Training}}$: Time required during the training of ML classifiers.

7 $$\begin{aligned} \text {Training Time} = T_{\text {preprocessing}} + T_{\text {clustering}} + T_{\text {training}} \end{aligned}$$

**Testing Time:** is the time required to process test samples using the trained ML model and produce a prediction. The testing time is calculated using Equation (8): Given that $\text {Start\_Time}_{\text {test}}$ is the time before the testing process begins and $\text {End\_Time}_{\text {test}}$ is the time recorded immediately after the testing process ends. The Python time module^38^ is employed to record execution time in milliseconds. Execution time may vary with hardware configuration, dataset complexity, and the nature of the applied ML algorithm.

8 $$\begin{aligned} \text {Testing Time} = \text {End\_Time}_{\text {test}} - \text {Start\_Time}_{\text {test}} \end{aligned}$$

**Memory Utilization During Training:** refers to the memory actively used in tasks, including dataset processing, clustering, and training ML models. The memory utilization during the training phase is calculated using Equation (9). Given that ${\text {Clustering}}$ is the memory required in clustering and labeling samples, ${\text {Preprocessing}}$ is the memory required for data preprocessing tasks, and ${\text {Training}}$ is the memory required during the training of ML classifiers. In this way, the equation provides a comprehensive view of the memory required for each component involved in the training phase of the ML model.

9 $$\begin{aligned} \text {Total Memory}_{\text {train}} = {\text {Clustering}} + {\text {Preprocessing}} + {\text {Training}} \end{aligned}$$

**Memory Utilization During Testing:** refers to the amount of memory required to process the test sample and produce predictions. The formula for memory utilization during the testing phase is given in Equation (10). Where $\text {Memory}_{a_{\text {test}}}$ is the memory usage just before the commencement of testing process, whereas $\text {Memory}_{b_{\text {test}}}$ is the memory utilization immediately after the testing process ends.

10 $$\begin{aligned} \text {Memory}_{\text {test}} = \text {Memory}_{a_{\text {test}}} - \text {Memory}_{b_{\text {test}}} \end{aligned}$$

**CPU Usage During Training:** During the training phase, the total CPU required for various processes. For example, CPU resources are needed to process input data, update centroids, and assign data points to clusters during the clustering process, data preprocessing utilizes the CPU to clean, normalize, and transform the dataset before training, and CPU is consumed during training the ML models. It can be calculated as given in Equation (11): Given that ${\text {Data\_labeling}}$ is the CPU utilization for labeling samples using clustering, ${\text {Preprocess}}$ CPU required for data preprocessing tasks and ${\text {Training}}$ CPU needed during the training of ML classifiers.

11 $$\begin{aligned} \text {Total CPU}_{\text {train}} = {\text {Data\_labeling}} + {\text {Preprocessing}} + {\text {Training}} \end{aligned}$$

**CPU Usage During Testing:** In the testing phase, the CPU is used to process test samples and predict results given in Equation (12). Given that $\text {CPU}_{a_{\text {test}}}$ is the CPU usage measured before the testing process starts and $\text {CPU}_{b_{\text {test}}}$ is the CPU usage measured immediately after the testing process ends. This study employs the ’psutil’ library to effectively monitor resource consumption during both the training and testing phases. The ’psutil’ is an open-source, cross platform python library^38^, designed to retrieve resource utilization and process management.

12 $$\begin{aligned} \text {CPU}_{\text {test}} = \text {CPU}_{a_{\text {test}}} - \text {CPU}_{b_{\text {test}}} \end{aligned}$$

## Performance evaluation of proposed SH-IDS

**Fig. 6:**
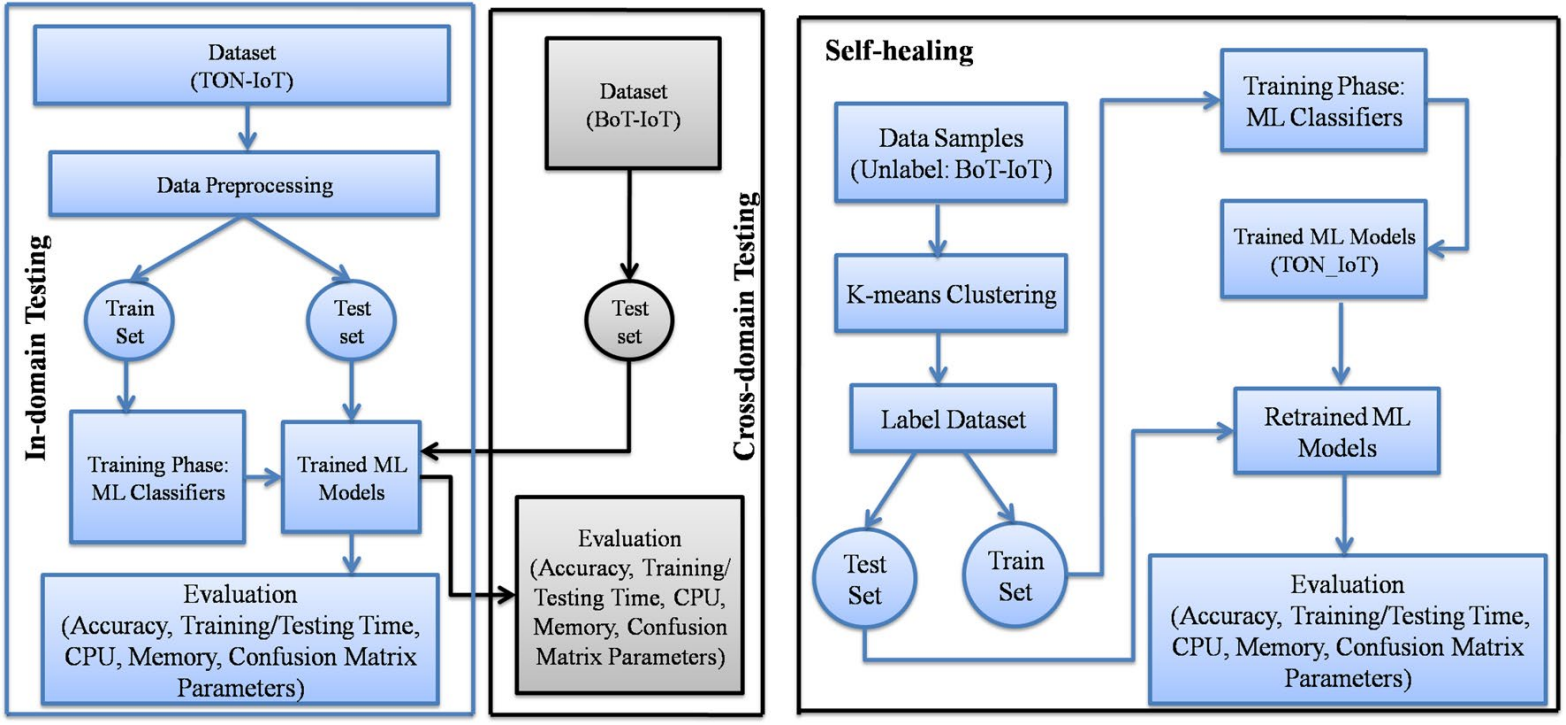
Evaluation flow for the proposed SH-IDS..

This section provides a comprehensive discussion of the experimental results to evaluate the proposed framework. The experimental flow is shown in Fig. 6; the TON_IoT dataset is used for in-domain testing, while the cross-domain evaluation is performed using the BoT-IoT dataset. The ML-enabled IDS models are evaluated using various performance metrics, including accuracy, training and testing time, CPU and memory usage, and confusion matrix parameters. The section consists of five parts: experimental setup, suitability of datasets for both in-domain and cross-domain testing, discussion of in-domain results, cross-domain evaluation, and evaluation of SH-IDS after self-healing.

### Experimental setup

In this section, we describe the experimental setup used to evaluate the proposed SH-IDS framework. All experiments are performed using Python 3.9.7 with Scikit-learn v0. 24. 2, Pandas v1. 3. 4, and NumPy v1. 20. 3 in the Anaconda environment^39^.Experiments are conducted on system with specifications including a single Intel Core i5-6600 CPU @ 3.30 GHz processor, 8 GB RAM, and MS Windows 10 Pro operating system. The Python psutil library is used to monitor resource measurements such as CPU usage, memory consumption, and training and testing time of each ML classifier.

### Contextual relevance of datasets

**Fig. 7:**
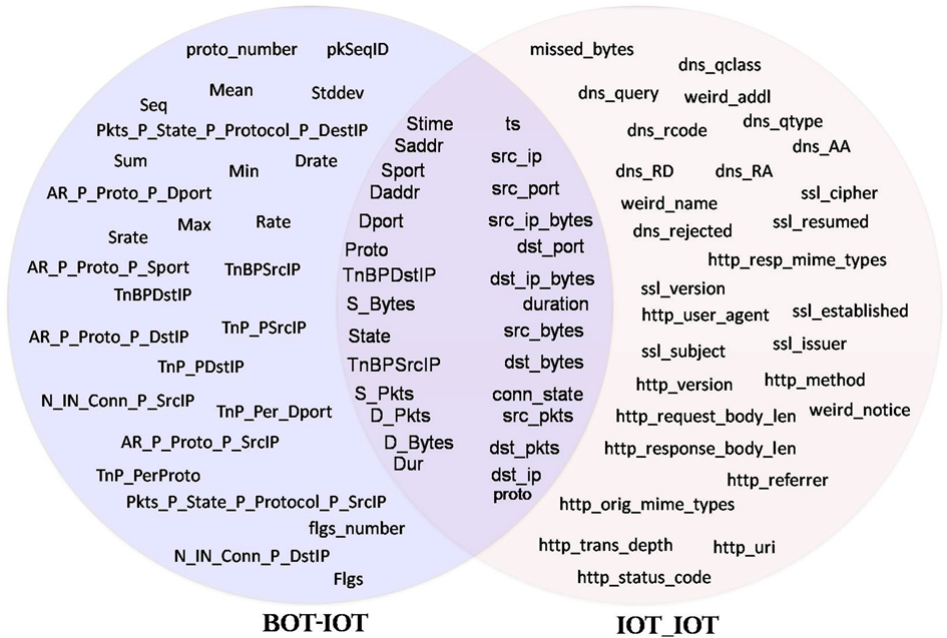
Features common in TON_IoT and BoT-IoT Datasets.

This section discusses the relevance between in-domain and cross-domain datasets. For in-domain testing, the TON_IoT dataset is used, while cross-domain evaluation is conducted using the BoT-IoT dataset. In this study, we selected the cross-domain dataset deliberately to be different from the training set but contextually similar, allowing us to evaluate the model’s performance in environments that share comparable characteristics. Notably, the cross-domain dataset is not provided to the model during training, which enables assessment of its generalization capability in real-world network conditions. The datasets chosen for this study satisfy the relevance criteria described in Table 4. Moreover, the feature sets in both datasets (TON_IoT and BoT-IoT) are highly similar for classifying attacks and normal network traffic, as illustrated in Figs. 7.

**Table 4:** Comparison of TON_IoT and BoT-IoT datasets.

Parameter	TON_IoT	BoT-IoT
Designed for IoT	Yes	Yes
Network traffic records	Yes	Yes
DoS/DDoS samples	Yes	Yes
Generated from testbed	Yes	Yes
Publicly available dataset	Yes	Yes
CSV and PCAP format	Yes	Yes

### In-domain results and discussion

This section discusses the results of in-domain testing. Data preprocessing substeps, as outlined in Sect. 4.1, are applied to the TON_IoT dataset. In subset extraction, 300,000 normal and 40,000 DoS/DDoS attack records are extracted from the TON_IoT dataset. Next, empty/null records are removed from the extracted subset. Undersampling is applied to the normal samples, resulting in 80,000 samples used for further experiments. Feature encoding is performed by converting categorical features into numerical values using label encoding. In addition, minimum scaling is applied to normalize the data by adjusting the feature range to [0, 1]. The preprocessed dataset is subjected to 10-fold cross-validation, which divides the dataset into training and testing sets. The training samples are provided as input to six ML classifiers: Random Forest (RF), Decision Tree (DT), Gradient Boosting (GB), K-Nearest Neighbor (KNN), Naive Bayes (NB), and Neural Network (NN) using the 14 features discussed in Sect. 2.3.

**Fig. 8:**
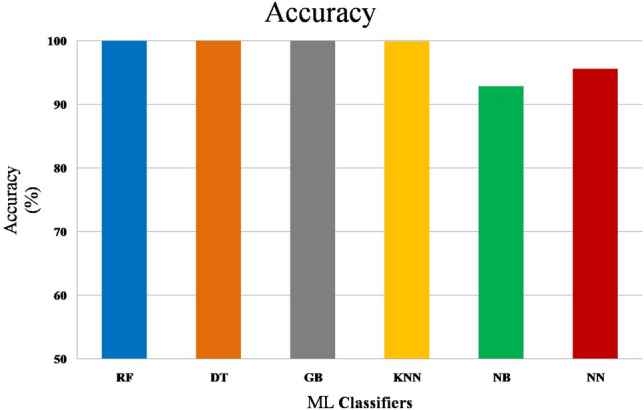
In-domain accuracy using TON_IoT dataset.

**Fig. 9:**
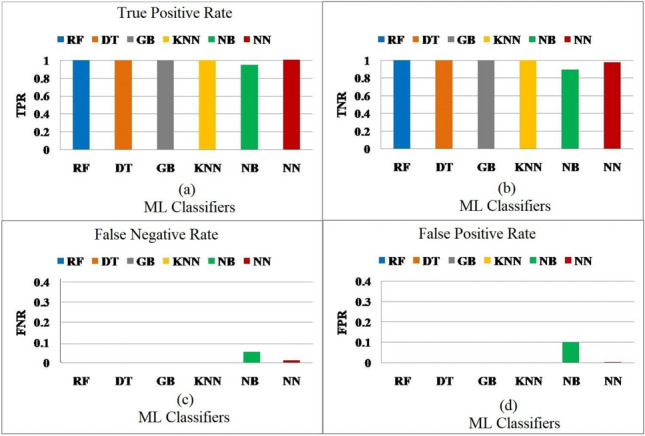
In-domain: Confusion Matrix using TON_IoT dataset.

The accuracy presented in Fig. 8 shows that the detection rates of RF, DT, GB, KNN, and NN classifiers are around 99.9%, which shows their ability to distinguish between normal and attack traffic. In comparison, the accuracy rate of the NB classifier was over 92%, although lower. However, it shows a satisfactory level of performance based on its probabilistic nature and statistical assumptions. Given in Fig. 9, ML classifiers including RF, DT, GB, KNN and NN achieve the highest TPR and TNR. However, the NB classifier exhibited a slightly higher FPR of 0.1 and produced negligible FNR, indicating minimal tendency to misclassify attack events.

**Fig. 10:**
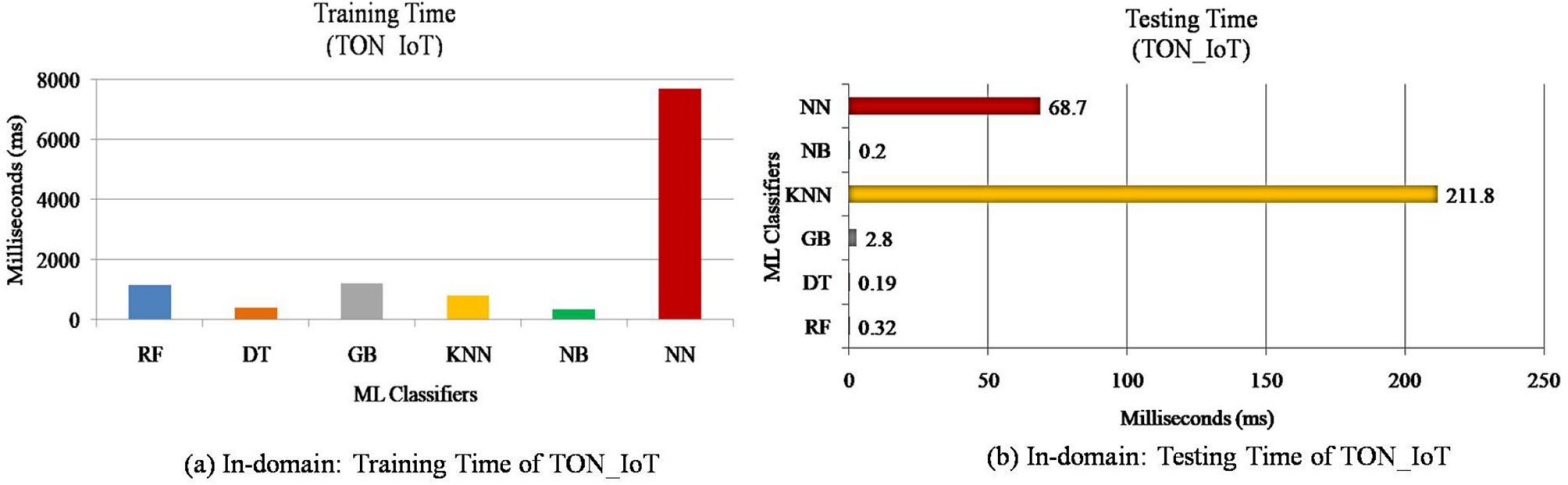
In-domain: Training and Testing Time.

As shown in Fig.  10 (a), NN requires the longest training time of 7,700 ms. The longest training time in NN reflects its complex architecture, which includes multiple layers, repeated weight adjustments, and extensive calculations. On the other hand, NB requires shortest time of 332 ms. NB follows a simple probabilistic approach with minimal processing. Figure  10 (b) shows the time required to test samples using the ML-enabled IDS model. The figure shows that KNN has the highest testing time among all ML classifiers, because KNN calculates the distance between each test sample and all training samples and then make decisions.

**Fig. 11:**
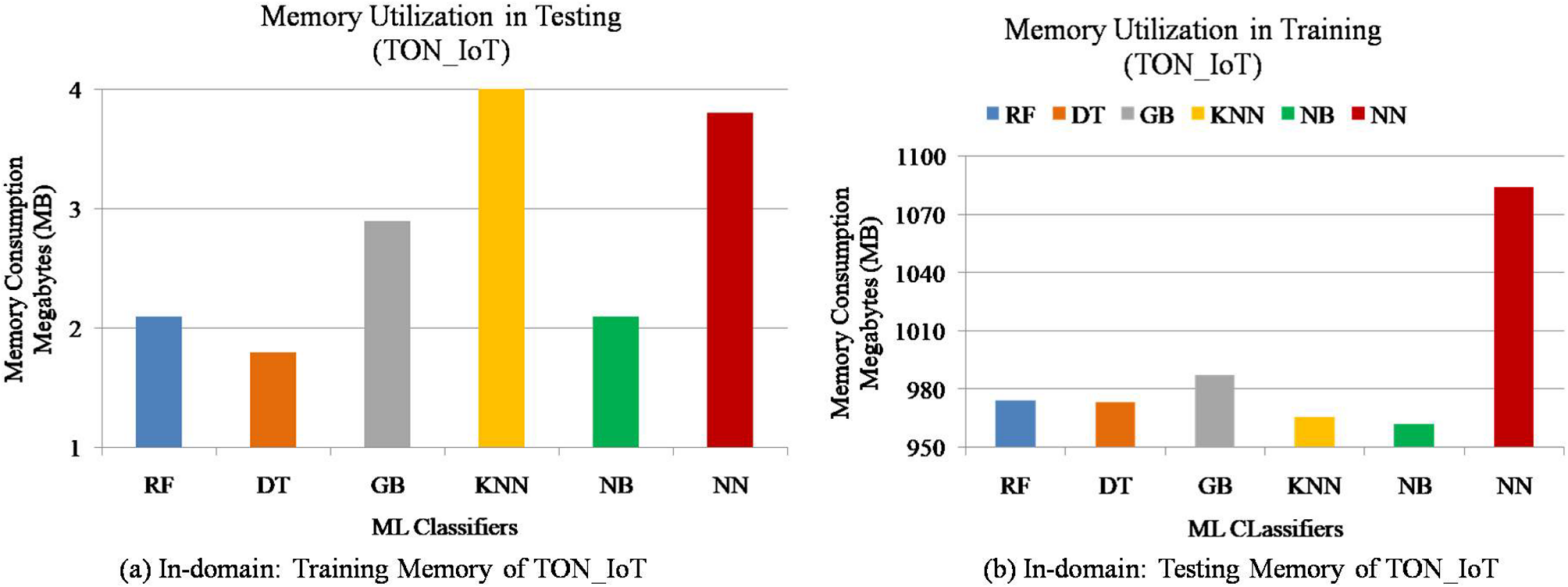
In-domain: Training and Testing Memory.

The memory usage during training, shown in Fig. 11a, NN requires the most memory of 1,083.98 MB. NB and KNN use only 962.3 MB and 965.8 MB, respectively. This is due to the complex architecture of NN, which involves storing multiple layers, weights, and activations, thus increasing the memory demand.

Given in Fig. 11b, KNN and NN consume maximum memory during testing, at 4 MB and 3.8 MB, respectively, whereas DT requires only 1.8 MB to process a single sample. This is because the KNN stores the entire training dataset for distance calculations, and the NN loads complex model parameters during inference.

**Fig. 12:**
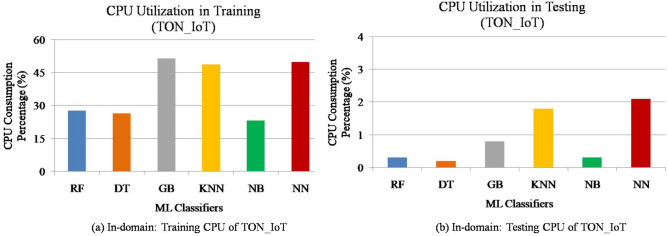
In-domain: Training and Testing CPU.

Figures  12a and b illustrate the CPU usage during training and testing. Figure 12a and b show that GB, NN, and KNN consume the most CPU as compared to other classifiers during training. KNN and NN maintain the highest CPU demand during testing. GB requires an iterative boosting process to build its knowledge profile, while NN and KNN are resource-intensive due to their complex internal structure. The in-domain results show that RF, DT, NN, GB, and KNN achieve the highest detection, while NB shows reasonable performance.

### Cross-domain results & discussion

This section presents a more rigorous cross-domain evaluation of the classification models. In this evaluation, samples from the BoT-IoT dataset are used to test the performance of ML classifiers trained on the TON_IoT dataset. Cross-domain evaluation provides insight into the generalization capability of the models, as the BoT-IoT samples are not used during training. In this section only the testing-phase results are presented. The data transformation steps for BoT-IoT follow the same preprocessing steps described in Section 4.1, and a total of 8,000 samples are selected for cross-domain evaluation.

**Fig. 13:**
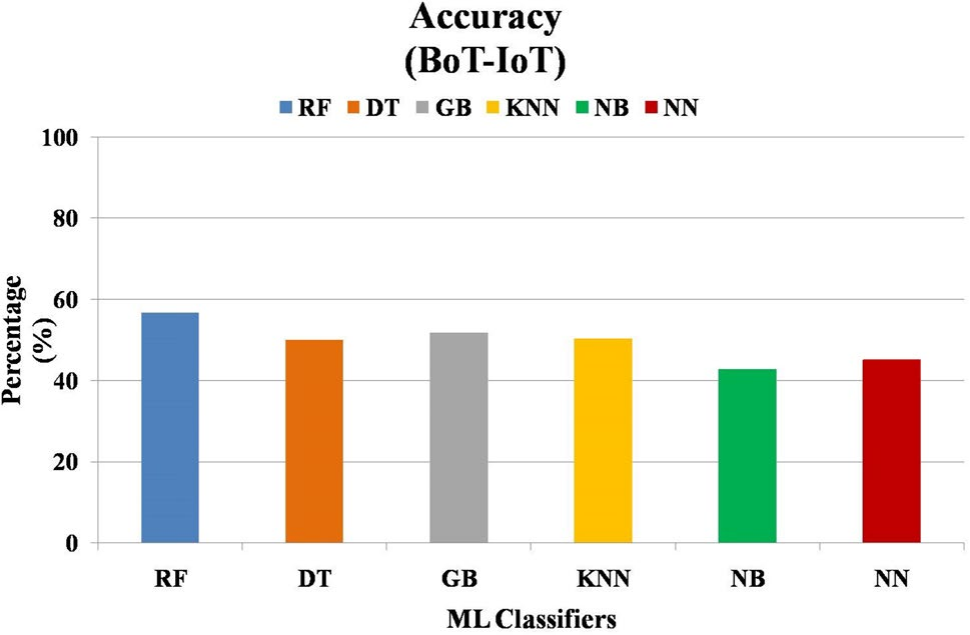
Cross-domain: Accuracy of BoT_IoT dataset.

Shown in Fig. 13, performance of ML classifiers significantly dropped in cross-domain testing compared to their performance in in-domain evaluations (see Fig. 8). Among all classifiers, the RF algorithm achieves the highest accuracy of 59.6%, and NB classifier produces the lowest accuracy of 41%. The lowest accuracy in NB is due to its reliance on probabilistic calculations, which makes it more sensitive to outliers.

**Fig. 14:**
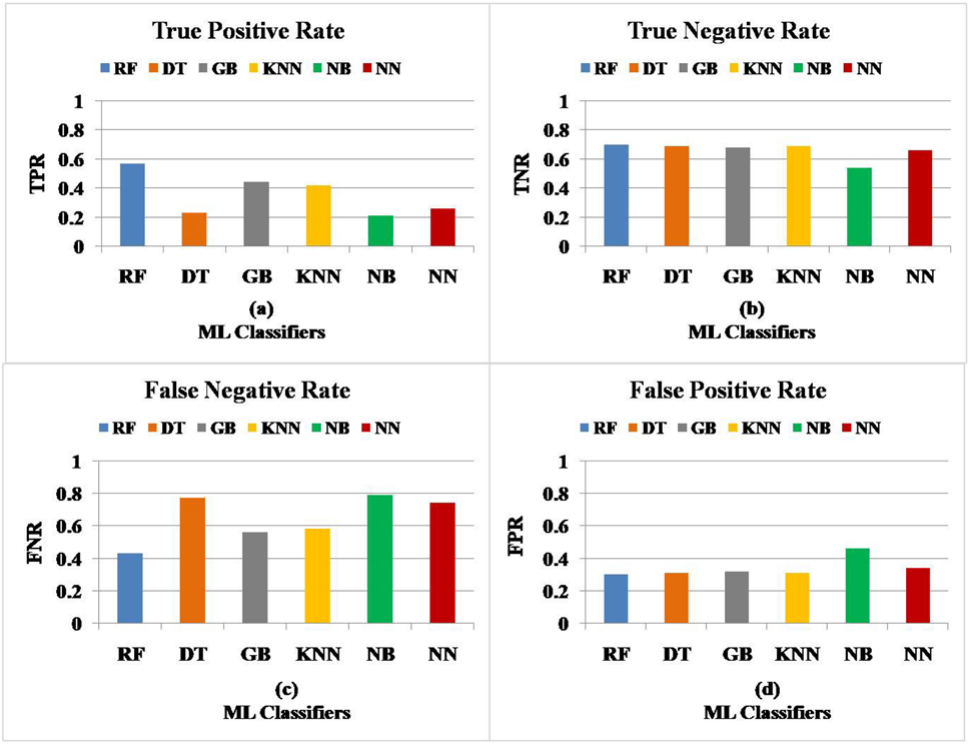
Cross-domain: Confusion Matrix of BoT-IoT dataset.

Similarly, depicted in Fig. 14, all ML classifiers, except NB, exhibit TNR value exceeding 0.6. Furthermore, the RF classifier achieves the highest TPR among all the classifiers, which shows its capability to identify attacks across different domains. The figure also illustrates that the DT and NB classifiers exhibit the highest FNR, indicating their limited capacity to generalize to unseen data. Additionally, the FPR value for the NB classifier is the highest among all the models considered. It can be attributed to the same feature independence assumption, causing it to misclassify negative instances as positive.

**Fig. 15:**
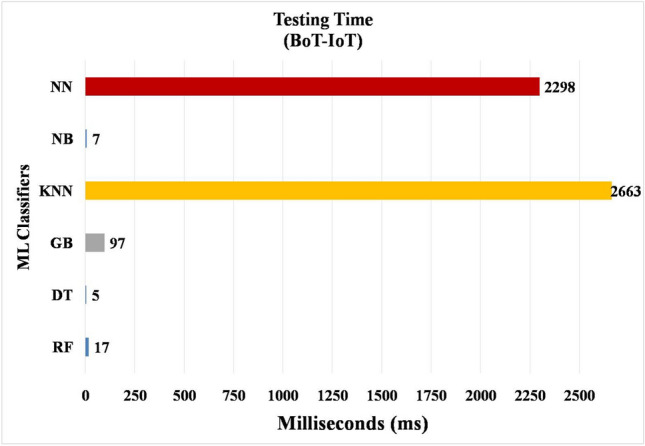
Cross-domain: Testing time of BoT-IoT dataset.

Figure 15 presents the testing time required to make predictions on 8,000 BoT-IoT data samples. In terms of testing time, both NN and KNN-based classifiers require significantly more time to test the considered samples due to their computationally intensive nature. However, the DT and NB classifiers perform best, with relatively low testing times of 5 and 7 msec, respectively.

**Fig. 16:**
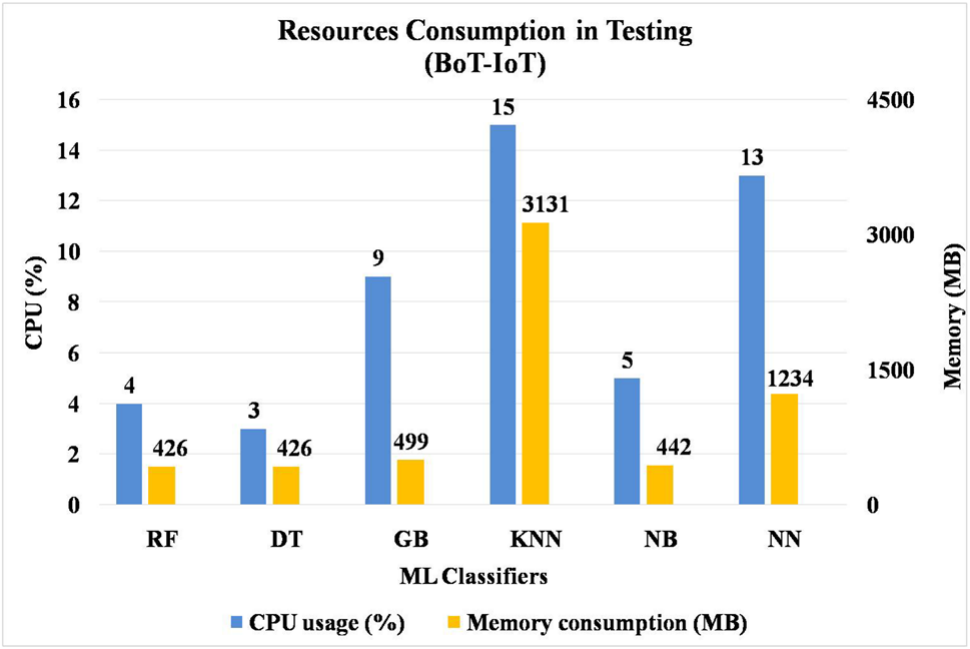
Cross-domain: Resource consumption of BoT-IoT dataset.

Moreover, Fig. 16 depicts the memeory and CPU consumption of six ML classifiers. In terms of memory consumption, DT, RF, and NB exhibit relatively low memory requirements. In contrast, KNN and NN exhibit high memory consumption when building the classification models. Regarding CPU usage, classifiers including RF, DT, and NB consume relatively low CPU resources (i.e., as low as 3%). Conversely, higher CPU usage of 15% and 13% is observed in the KNN and NN classifiers, respectively.

The cross-domain evaluation in figures from (Figs.13 to 16) show that the RF classifier achieves the best overall performance, with 59.6% accuracy, a TPR of 0.57, and an FNR of 0.43. In addition, RF requires only 0.15 MB of memory, 0.00037% CPU, and 0.0007 ms per test sample, making it the most efficient choice. Despite in decline in accuracy, the IDS maintains over 50% detection performance in a completely new environment, demonstrating robustness against previously unseen data.

### Evaluation after self-healing

In this experiment, the BoT-IoT dataset is used to extend the analysis presented in Sect. 5.4. IDS models trained on the TON_IoT dataset and tested on the BoT-IoT dataset showed performance degradation during cross-domain assessment as shown in Fig. 13. To detect attacks and enhance the robustness of the IDS. We continue the assessment using the BoT-IoT dataset given in the following sections.

The self-healing process is initiated upon the generation of a threat signal. As shown in Fig.  18, the packet rate limit is set to 20.4 ms, the CPU limit to 21 ms, and the memory limit to 24 ms. The threat signal is activated only when all resources hit their respective threshold values. This requires a multifactor analysis that considers multiple variables to generate the threat signal. Considering one variable does not always indicate that a DoS/DDoS attack is imminent. Therefore, in this study, the self-healing process is initiated based on the individual thresholds of the three factors.

**Fig. 17:**
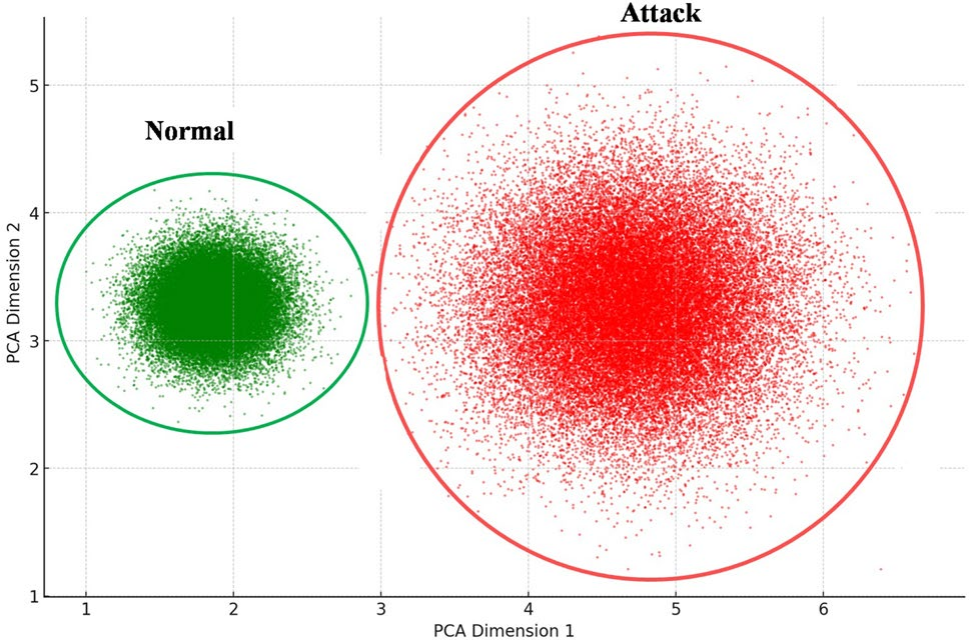
Normal and attack clusters..

To test the performance of K-means clustering, we used the PCAP files of the BoT-IoT dataset. These files contain both attack and normal traffic records. We created a dataset of 80,000 records, in which 40,000 represent normal traffic, and 40,000 represent DoS/DDoS attack traffic. The selected samples from the PCAP files are converted to CSV format, preserving 38 features, which serve as input to the K-means clustering algorithm. As shown in Fig 17, Clustering and labeling are performed based on the timestamp of the danger signal generated after the clustering (discussed in Section IV-D).

**Fig. 18:**
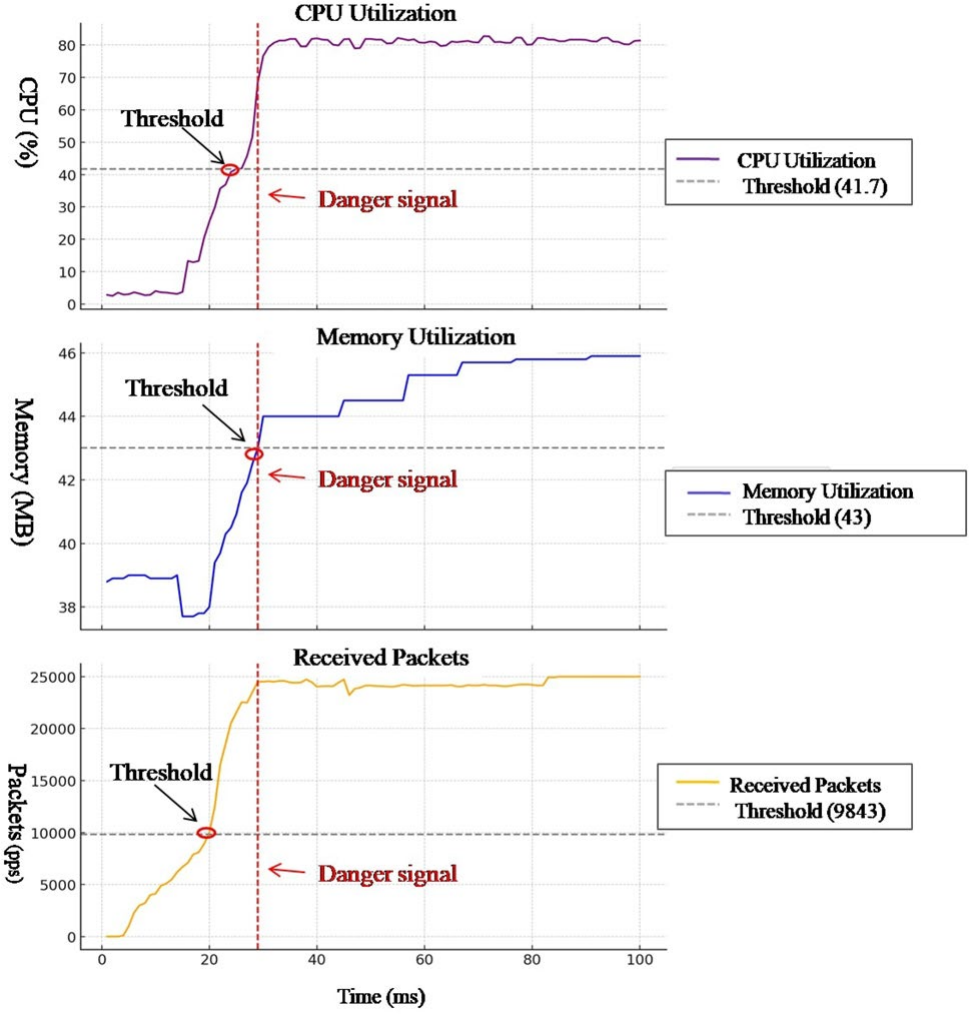
Danger signal generation in response to threshold activation..

The output dataset produced after clustering and labeling consists of 39 features. The dataset contains all the packet features along with an additional feature, ‘label’, which designates each sample as ‘normal’ or ‘attack’. This labeled dataset serves as the key input for the supervised ML classifiers during the training of the IDS model.

The retraining process requires pre-trained ML models and a labeled dataset. First, data preprocessing steps (described in section  4.1) are applied to the data to remove missing and incomplete records. Next, the pre-processed dataset is split into training and testing samples using 10-fold cross-validation. The training samples are given as input to train the ML classifiers. It is worth noting that the IDS model integrates the new learning of the ML classifiers with their previous learning after retraining them. This ensures that the updated IDS models are not only capable of detecting new attacks, but also of detecting existing attacks.

**Fig. 19:**
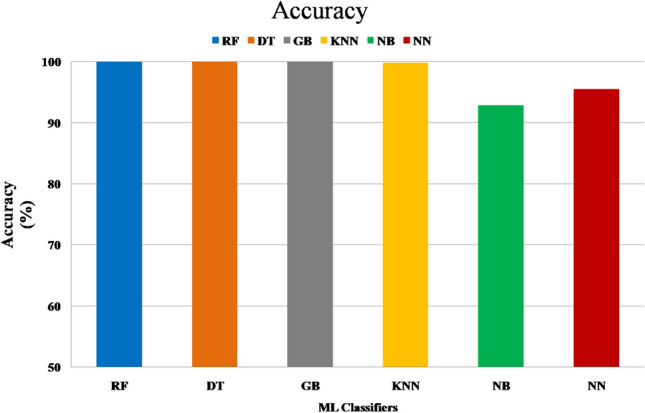
Accuracy after self-healing using BoT-IoT dataset.

The accuracy presented in Fig. 19 shows a substantial improvement in ML model performance after retraining. Compared to the results presented in Fig. 13. Illustrated in Fig. 19, RF, GB, and DT achieve accuracy of 99.9%, a significant increase from their previous scores of 59.6%, 54.9%, and 50.6%, respectively. The performance of the classifiers demonstrates that retraining significantly enhances model generalization across domains, confirming its effectiveness in improving IDS performance in diverse IoT environments.

**Fig. 20:**
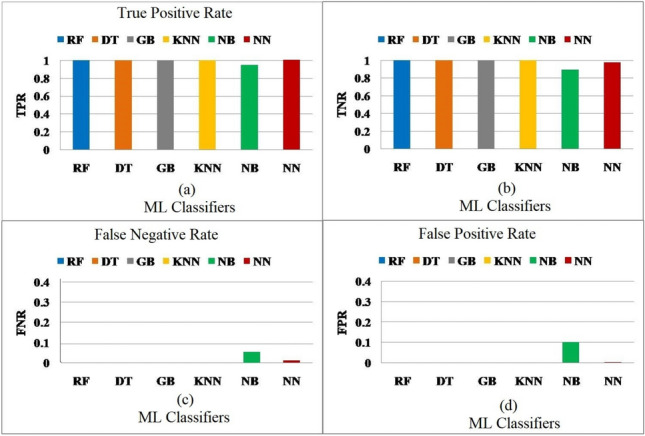
Confusion matrix of BoT-IoT dataset after self-healing.

Figure 20a–d illustrate the TPR, TNR, FNR, and FPR values for ML classifiers. As shown in Fig. 20a, RF, DT, KNN, and GB achieve a highest TPR of 1. Figure 20b presents that ML models achieve TNR of 1. In contrast, NN and NB show slightly lower performance, with TNR values of 0.9 and FNRs of 0.1 , respectively depicted in Fig. 20c. Figure 20d further reveals that NB and NN record the highest FPR (0.17 and 0.02). Overall, RF, DT, KNN, and GB demonstrate consistently superior detection and effectively distinguishing between normal and attack traffic.

**Fig. 21:**
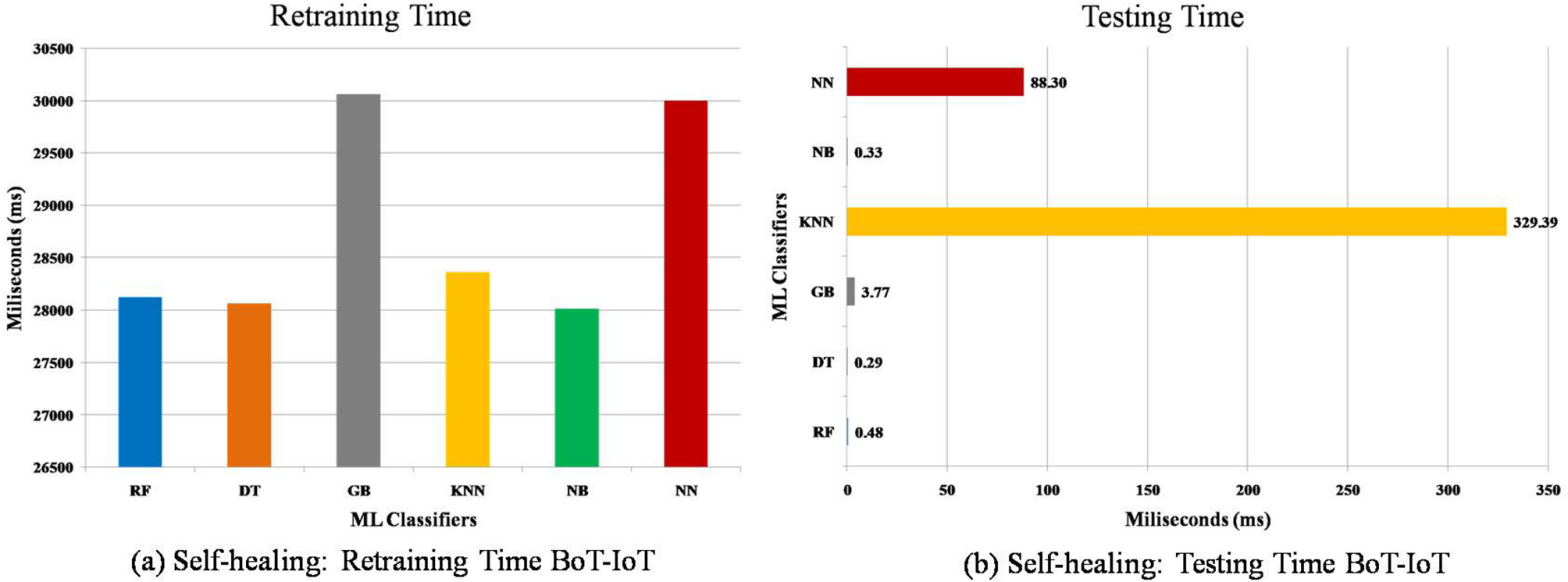
Retraining and Testing time after self-healing.

Figure 21a illustrates the training times required for six ML classifiers employing 14 features (shown in Fig. 7) from the BoT-IoT dataset.

Figure 21a shows the retraining times of the ML classifiers. The NB model demonstrates the shortest retraining of 28,013.3 ms. Whereas, DT and RF models require 28,063.9 ms and 28,120 ms, respectively. In contrast, GB and NN exhibit the highest retraining times, approximately 30,000 ms. GB uses an ensemble learning approach to build its knowledge base and NN with multi-layer structure requires intensive computational cost^40^.

Figure 21b presents the testing times for evaluating the individual BoT-IoT samples. Among all classifiers, DT achieves the fastest prediction speed, processing each sample in just 0.29 ms. NB and RF follow with slightly higher testing times of 0.33 ms and 0.48 ms, respectively. Conversely, KNN records the longest testing duration of 329.39 ms.

**Fig. 22:**
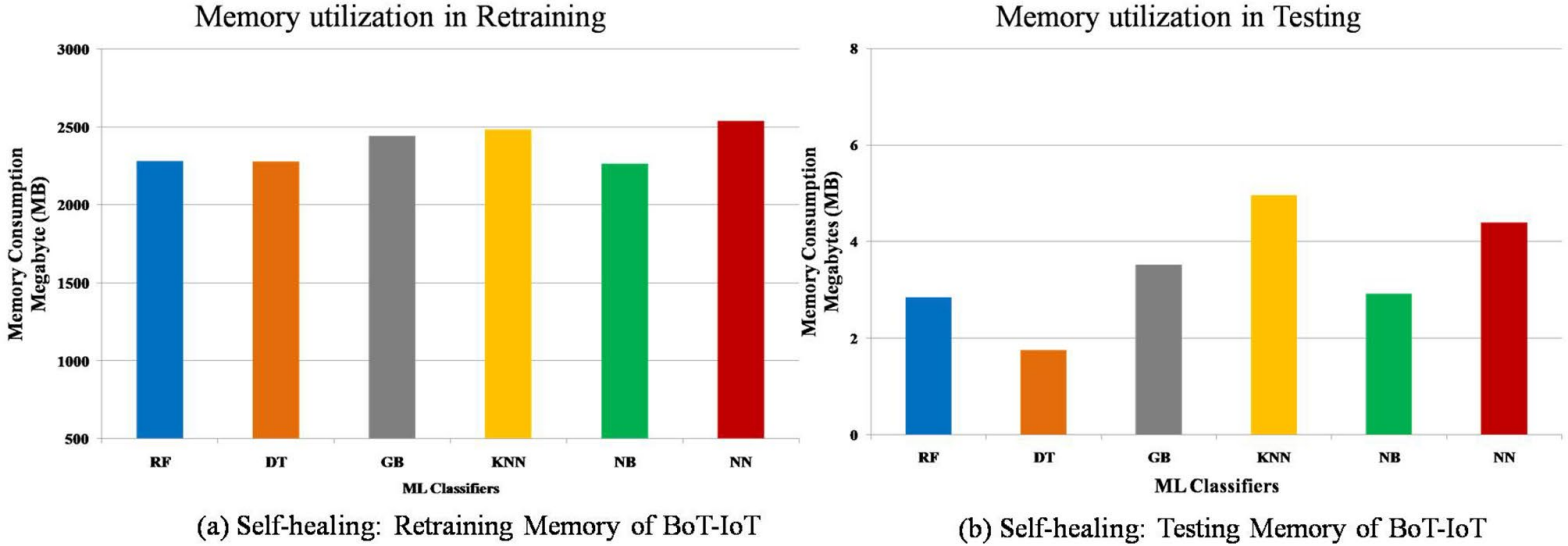
After self-healing: Retraining and Testing memory.

Figure 22a illustrates the memory requirements during retraining. According to the results, the NB has the lowest memory consumption, requiring only 2264 MB, which reflects its relatively simple probabilistic architecture that demands fewer computational resources. Following NB, RF, and DT consume slightly higher memory, of 2280 MB and 2278 MB, respectively. In contrast, NN shows the highest memory consumption, reaching 2539 MB.

Figure 22b shows the memory consumption of each trained ML model during the testing phase. The results indicate that all models, except KNN, exhibit moderate memory usage. However, KNN exhibits the highest memory consumption at 4.96 MB. Conversely, the RF, DT, GB, and NB classifiers demonstrate the lowest memory usage during testing, each requiring less than 4 MB for testing a BoT-IoT sample.

**Fig. 23:**
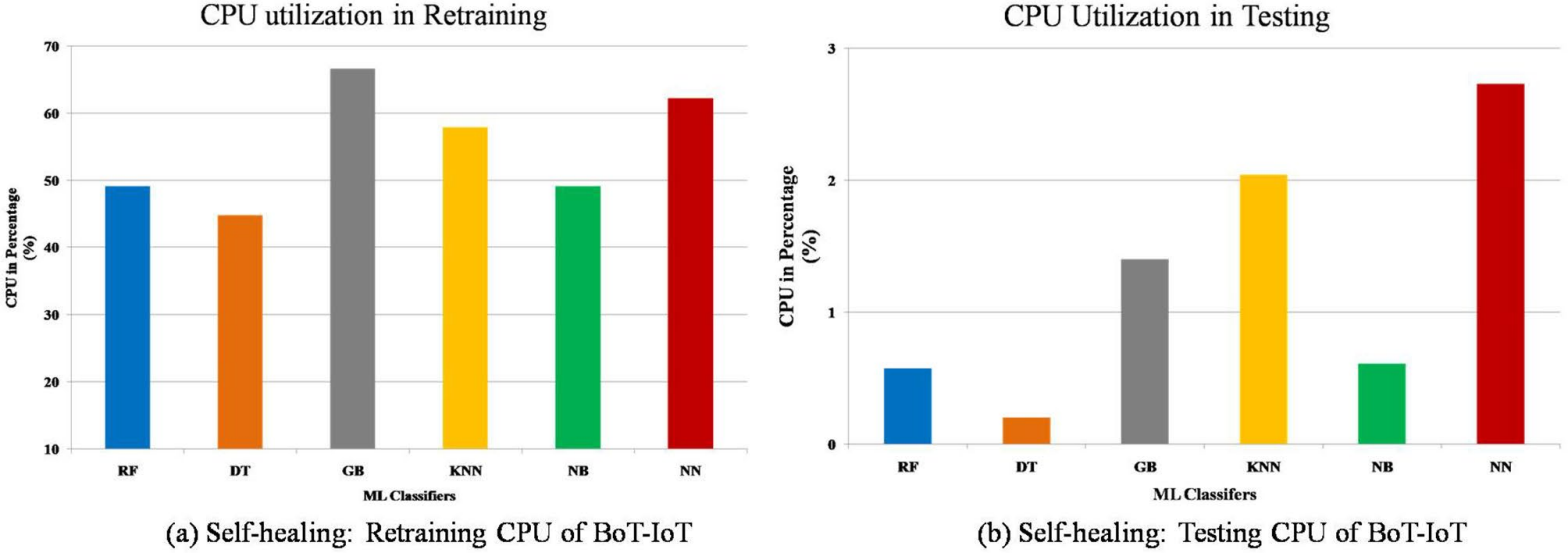
After self-healing: Retraining and Testing CPU .

The CPU usage during the retraining phase for the BoT-IoT dataset is shown in Fig. 23a, which shows that the DT model requires the lowest CPU consumption approximately 45%. In contrast, RF and NB second lowest CPU consumption about 45%. In contrast, GB and NN exhibit the highest CPU utilizations 66.6% and 62%, respectively. During the testing phase, as given in Fig. 23b. DT uses the least amount of CPU of 0.2%, followed by RF at 0.58%. On the other hand, KNN and NN, have the highest CPU utilization of up to 2.73%.

Overall, the results presented in Figs. 19 to 23 summarize the model accuracy, confusion matrix parameters, and resource consumption in time, memory, and CPU. The evaluation shows that after self-correction, IDS models including RF, DT, GB, and KNN provide the highest accuracy and effectively distinguish between benign and invasive traffic. In terms of resource efficiency, DT, RF, and NB show the lowest usage during both retraining and testing, while KNN, GB, and NN show higher demands.

Table 5 presents a comparison between peak accuracies of existing state-of-the-art with this study. This shows the overall correctness of the models in detecting malicious and benign traffic collectively. It is evident from the table that our approach dominates other studies with a peak accuracy of 99.9%. on the other hand, with other approaches having lower accuracy are prone to attacks. In this study, we have also calculated the TPR to specifically evaluate the attack detection rate. Since, if undetected, an attack packet may compromise the overall security of the network. Moreover, our study evaluated several other essential key performance indicators including all parameters of confusion matrix, CPU/memory usage and time consumption which is lacking in previous studies. Consequently, giving a complete picture of the performance and making SH-IDS an efficient and lightweight solution suitable for resource-constrained IoT environments.

**Table 5:** Comparison of Peak Accuracy with Existing Studies.

Paper ID	Peak Accuracy (%)
^24^	83
^27^	85
^26^	88
^11^	90
^25^	91.5
^9^	94
^10^	95
^21^	95.3
^22^	97
^23^	99
**This Study**	**99.9**

## Conclusions

This paper provides a comprehensive discussion on the increasing prevalence of DoS/DDoS attacks and the shortcomings of current state-of-the-art IDS approaches in securing IoT-based systems. To address these limitations, this study presents a novel framework *SH-IDS* that leverages ML intelligence and incorporates a self-healing mechanism to effectively detect existing and emerging DoS/DDoS attacks. The self-healing capability of SH-IDS employs a collaborative approach, where IoT devices running the health module are responsible for triggering the self-healing process in the IDS. The resource-centric triggering mechanism protects IoT devices from failure, as the healing process is activated only when resource usage exceeds predefined thresholds. Moreover, the event-driven nature of the mechanism prevents system overload by ensuring adaptation occurs only when necessary. This self-healing capability enables the IDS to detect new attacks in real-time. *SH-IDS* is evaluated using six ML classifiers using TON_IoT and BoT-IoT datasets to demonstrate its effectiveness in both cross-domain and in-domain scenarios. The evaluation results show that, after the self-healing process, *SH-IDS* increases its accuracy in detecting DoS/DDoS attacks with RF by 40.3% and significantly reduces false alarms. *SH-IDS* is further evaluated for resource consumption, which shows that *SH-IDS* has a latency of 0.47 ms, memory usage of 3.3 MB, and CPU usage of 0.71% per sample. Moreover, with the IDS requiring 697 KB of storage, this analysis shows that the proposed framework is efficient in terms of responsiveness and resource usage. Our future work will optimize *SH-IDS* for low-power IoT devices using lightweight ML models, evaluate it in real-world environments, and investigate robustness against concept drift and adversarial attacks.

## Data Availability

Data are available from the corresponding author upon reasonable request.
